# Pathogenicity and Long-Term Outcomes of Liddle Syndrome Caused by a Nonsense Mutation of *SCNN1G* in a Chinese Family

**DOI:** 10.3389/fped.2022.887214

**Published:** 2022-05-24

**Authors:** Di Zhang, Yi Qu, Xue-Qi Dong, Yi-Ting Lu, Kun-Qi Yang, Xin-Chang Liu, Peng Fan, Yu-Xiao Hu, Chun-Xue Yang, Ling-Gen Gao, Ya-Xin Liu, Xian-Liang Zhou

**Affiliations:** ^1^Department of Cardiology, National Center for Cardiovascular Diseases, Chinese Academy of Medical Sciences and Peking Union Medical College, Fuwai Hospital, Beijing, China; ^2^Emergency and Critical Care Center, National Center for Cardiovascular Diseases, Chinese Academy of Medical Sciences and Peking Union Medical College, Fuwai Hospital, Beijing, China; ^3^Department of Geriatric Cardiology, Chinese People’s Liberation Army (PLA) General Hospital, Beijing, China

**Keywords:** Liddle syndrome, pathogenicity, pediatrics, amiloride-sensitive current, longterm prognosis

## Abstract

**Objective:**

Liddle syndrome (LS) is a monogenic hypertension consistent with autosomal dominant inheritance, often with early onset high blood pressure in childhood or adolescence. This study aimed to identify the pathogenicity of a nonsense mutation in *SCNN1G* in a Chinese family with LS and the long-term outcomes of tailored treatment with amiloride.

**Methods:**

To explore the pathogenicity of candidate variant reported in 2015 by our team, we constructed mutant and wild-type models *in vitro* and measured amiloride-sensitive current in *Chinese Hamster Ovary* (*CHO*) cells using patch clamp technique. Participants were followed up for 7 years after tailored treatment with amiloride.

**Results:**

A nonsense variant was detected in six members, two of whom were pediatric patients. This mutation resulted in a termination codon at codon 572, truncating the Pro-Pro-Pro-X-Tyr motif. The mutant epithelial sodium channels displayed higher amiloride-sensitive currents than the wild-type channels (*P* < 0.05). Tailored treatment with amiloride achieved ideal blood pressure control in all patients with normal cardiorenal function, and no adverse events occurred during follow-up.

**Conclusion:**

We found the pathogenicity of a nonsense *SCNN1G* mutation (p.Glu571*) with enhanced amiloride-sensitive currents in a LS family with young patients. Tailored treatment with amiloride may be an effective strategy for the long-term control of blood pressure and protection from target organ damage or cardiovascular events, including children and youth patients with LS.

## Introduction

Liddle syndrome (LS) was first reported at 1963 by Liddle et al. (1). It is a monogenic hypertension caused by mutations in genes encoding epithelial sodium channels (ENaCs) (2). ENaCs are composed of three subunits: α, β, and γ (encoded by *SCNN1A*, *SCNN1B*, and *SCNN1G*, respectively) (3). Proline-rich segments, referred to as Pro-Pro-Pro-X Tyr (PY) motifs, in the carboxyl terminal regions of β and γ subunits are binding sites for WW domains of neural precursor cell expressed, developmentally Down-regulated 4 (NEDD4), an E3 ubiquitin-protein ligase (4). By binding the ubiquitin-protein ligase domain, PY motifs inactivate ENaCs (5). LS is characterized with increased sodium reabsorption in collecting tubules resulted from ENaCs dysfunction, including inactivation disorders, and abnormal opening frequencies (3, 6–8). The function of ENaCs usually is examined by patch clamp techniques.

The typical manifestations of LS include early-onset hypertension, hypokalemia and suppression of plasma renin activity (PRA), and plasma aldosterone concentration (PAC) (3, 9). Some LS patients lack a typical presentation and are not diagnosed and treated in time, which can lead to major complications such as stroke and early death because of poor control of hypertension (10–13). Genetic sequencing is recommended for LS screening for *SCNN1A*, *SCNN1B*, and *SCNN1G* mutations, especially in families with a history of early-onset hypertension (3). Through family screening, it often screens for children who carried mutations in family (11, 14). Genetic sequencing screened LS patients, but the pathogenicity of most variants has not been verified by functional experiments. For LS patients diagnosed based on sequencing results and silico analysis prediction, the usual follow-up after beginning amiloride treatment is approximately 1 or 2 months and the long-term outcomes of LS patient remains unclear.

In this study, we explored the pathogenicity of a nonsense mutation (c.1171G > T) in exon 13 of *SCNN1G* reported in 2015 year by our team and efficiency of blood pressure control by amiloride. We used patch-clamp to verify functional mechanism of this mutation, and found that amiloride-sensitive currents differed significantly between wild-type and mutant groups. All members harboring the c.1171 G > T mutation in *SCNN1G* with hypertension and/or abnormal biochemical presentations were diagnosed with LS. Over a 7-year follow-up, the results show efficacy with tailored treatment of amiloride in blood pressure control and prevention of target organ damage.

## Materials and Methods

### Clinical Characteristics

#### Subjects

The index case was an 18-year-old woman (III-2) with early-onset, refractory, and unexplained hypertension and hypokalemia, accompanied by suppressed PRA. The diagnosis of hypertension for children is made when repeat BP values on three different measurements are greater than the 95th percentile for the age, sex, and height of the patient (15). The proband and five members carrying the nonsense mutation were included for the clinical, biochemical analysis in this family. This study was approved by the Ethics Committee of Fuwai Hospital and all participants signed their written informed consent (The informed consent of the deceased patient was obtained from his immediate family) in accordance with the Declaration of Helsinki.

#### Tailored Treatment and Follow-Up

All biochemical tests, such as of electrolytes, were performed using standard methods. Patients carrying *SCNN1G* mutation were required to follow a salt-restricted diet and received oral amiloride (5.0 mg/day for adults and 2.5 mg/day for children) intervention. Blood pressure and potassium levels were measured during follow-up period. Indicators of assessing cardiorenal function and cardiovascular events were followed up over 7 years. Indicators included creatinine, urea nitrogen, echocardiography, and cardiovascular events. Events contain heart failure, stroke, and all-cause death.

#### Functional Analysis of the γG571 Stop Mutation in Epithelial Sodium Channels

##### Site-Directed Mutagenesis and Chinese Hamster Ovary Cell Incubation

The sequence of human *SCNN1A*, *SCNN1B*, and *SCNN1G* was obtained from the University of California Santa Cruz database, and cDNAs encoding human ENaC were synthesized exogenously. The *SCNN1G* gene mutation (c.1171G > T) was induced using a QuikChange Site-Directed Mutagenesis Kit (Statagene). ENaC expression vectors were gifts from Dr. Cecilia Canessa, Yale University. *Chinese Hamster Ovary* (*CHO*) cells were used for expression human ENaC. *CHO* cells were cultured under standard conditions (10% fetal bovine serum in Dulbecco’s modified Eagle’s medium, 1% penicillin/streptomycin, 37°C, 5% CO_2_). Before transfection, cells were seeded on poly-lysine coated slides. pcDNA3.1-ENaCα, ENaCβ, ENaCγ, or ENaCγ-mut together with pEGFP-N1 were transfected into cells in a 1:1:1:1 ratio using Lipofectamine 2000 (Invitrogen, Carlsbad, CA, United States). Green fluorescent protein was used to identify transfected cells. Amiloride (10 μM) was added to culture medium until just prior to the electrophysiological experiments (48 h after transfection).

##### Electrophysiological Measurements

Whole-cell current recordings were acquired using a MultiClamp 700B amplifier (Molecular Devices, San Jose, CA, United States). Signals were collected by a Micro 1401 MKII (Cambridge Electronic Design, Cambridge, United Kingdom) using Spike 2 acquisition software. The electrophysiological methods were as previously described (16). In brief, the pipette solution (in mM) was 120 CsCl, 5 NaCl, 5 EGTA, 2 MgCl_2_, 2 Mg-ATP, 0.1 GTP, and 10 HEPES (pH 7.4). The bath solution (in mM) was 150 NaCl, 1 CaCl_2_, 2 MgCl_2_, and 10 HEPES (pH 7.4). Whole-cell capacitance was recorded for normalizing ENaC currents. Current through ENaC was elicited by voltage ramping from 20 to -140 mV over a 300 ms period. At the end of each recording, 10 μM amiloride was added to identify amiloride-sensitive currents. ENaC activity was assessed as the amiloride-sensitive current.

##### Systematic Review of Published Studies Regarding Liddle Syndrome Patients Carrying SCNN1G Mutations

We systematically searched MEDLINE (*via* PubMed), Embase, Cochrane, and Web of Science databases for relevant articles published in English up to December 31, 2020, with the search terms “Liddle syndrome,” “Liddle’s syndrome,” “pseudoaldosteronism,” and *SCNN1G*. Studies that reported patient clinical characteristics, outcomes as well as phenotype and genotype relation of Liddle syndrome patients carrying *SCNN1G* mutations and pathogenicity of variants were included.

### Statistical Analysis

Differences in amiloride-sensitive sodium currents between wild-type and mutant ENaCs in *CHO* cells were analyzed using the unpaired *t*-test. *P-*values less than 0.05 were considered statistically significant.

## Results

### Clinical and Biochemical Characteristics

All members participating in this study presented with varying degree of hypertension. The echocardiographic findings of the proband (III-2) suggest concentric left ventricular hypertrophy, so it is presumed that hypertension occurred earlier than 18 years old. The I-1 and II-2 were paralyzed and bedridden from hypertensive cerebral hemorrhage prior the start of the study and the II-3 was diagnosed with hypertension and died of hypertensive stroke at 32 years of age. The IV-1, a 7 years old boy, was diagnosed with hypertension for BP values (120/72 mmHg) greater than 95th percentile reference (110/71 mmHg). All participants underwent biochemical examinations, and the results are shown in Table 1.

**TABLE 1:** Clinical, biochemical characteristics and results of follow-up of patients in this family.

Cases	Sex	Age (years)	Max BP (mmHg)	Serum K + (mmol/L)	Follow-up at 1 month			Follow-up at 7 years							
					BP (mmHg)	Serum K + (mmol/L)	BP (mmHg)		Serum K + (mmol/L)	Creatinine (μmol/L)	Urea nitrogen (mmol/L)	LVEDD (mm)	IVS thickness	LVEF (%)	Events
I-1	F	78	176/106	2.83	134/78	4.22	NA		NA	NA	NA	NA	NA	NA	No
II-1	F	58	194/116	2.76	126/80	4.59	110–130/82–90		3.62–4.9	89.68	6.20	60	11	55	No
II-2	M	54	210/120	2.00	130/74	4.22	128–136/78–84		4.51–4.66	74.41	5.20	49	12	69	Previous Stroke
II-3	M	32	NA	NA	NA	NA	NA		NA	NA	NA	NA	NA	NA	Death
III-1	F	31	140/110	2.62	138/76	3.92	112–129/80–92		3.80–4.55	56	2.92	40	10	70	No
III-2	F	18	216/118	2.50	126/80	4.24	120–130/85–90		3.45–4.12	62	3.02	45	13	72	No
IV-1	M	7	120/72	3.32	NA	NA	102–120/60-80		3.56–4.0	108	4.85	43	10	60	No

*F, female; M, male; BP, blood pressure; the reference of serum K^+^: 3.5–5.5 mmol/L; the reference of creatinine: 44–133 μmol/L; the reference of urea nitrogen: 2.86–7.9 mmol/L; LVEDD, left ventricular end-diastolic dimension; IVS, interventricular septum; LVEF, left ventricular ejection fraction; NA, not available.*

### Tailored Medicine for Mutation Carriers

The proband and other members diagnosed with LS were recommended to follow a low-salt diet and receive oral amiloride treatment. The blood pressures and serum potassium levels of all family members were in the normal range (Table 1) during follow-up period. Creatinine, urea nitrogen and the result of echocardiography were normal, and none of the family members had cardiovascular events during the 7-year period of regular treatment (Table 1).

### Electrophysiology

**FIGURE 1:**
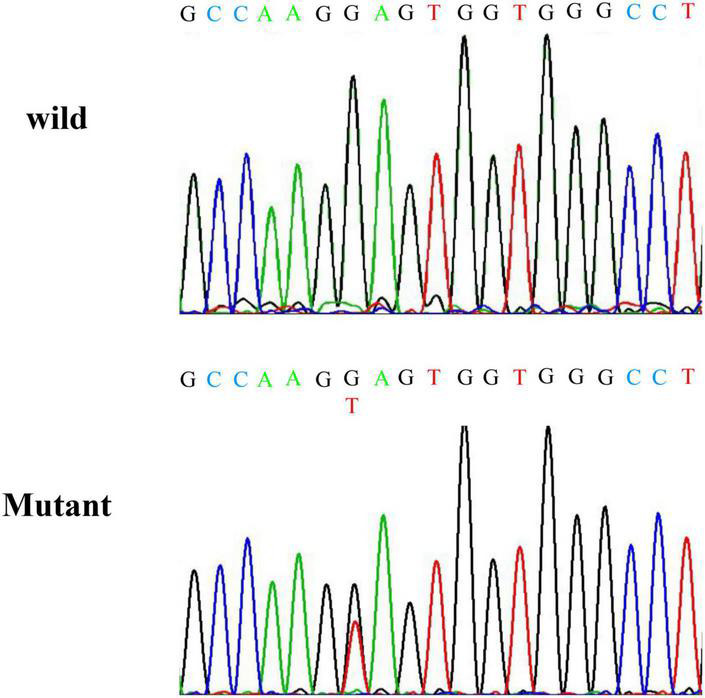
Results of Sanger sequencing. Sanger sequencing indicates a nonsense mutation at codon position 562 of exon 13 of *SCNN1G.*

**FIGURE 2:**
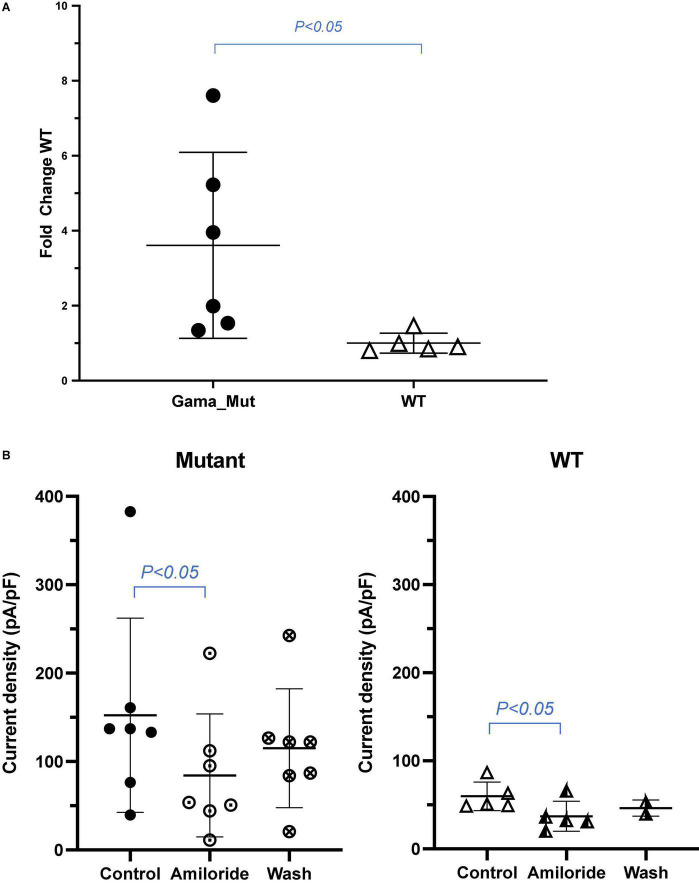
**(A)** Sodium current between wild and mutant group. Amiloride-sensitive sodium current was increased in CHO expressing mutant ENaC compared with those expressing wild type channels. Values are significantly different between wild type. **(B)** Amiloride inhibition of sodium current in wild and mutant group. Amiloride-sensitive sodium current was decreased in *CHO* of mutant ENaC after adding amiloride compared with controlled group. Values are significantly different between with amiloride and without amiloride (*P* < 0.05).

A truncation mutation in exon 13 of *SCNN1G* was identified and reported in this family and this mutation led to a stop codon after glutamic acid (Glu) (p.Glu571*; c.1171G > T; Figure 1). We compared amiloride-sensitive sodium currents in p.Glu571* mutant and wild-type *CHO* cells. The amiloride-sensitive currents of the mutant control group were 3.7 times of the wild-type control group (3.7 vs. 1.0, *P* < 0.05; Figure 2A). After cells were incubated for 48 h after the addition of amiloride, we detected and compared the current changes in wild-type and mutant ENaCs cells. There was a significant difference between the mutant control and mutant amiloride-added groups (152.4 ± 41.6 pA/pF vs. 84.2 ± 26.3 pA/p, *P* = 0.013), indicating that the sodium current was inhibited by amiloride, an ENaC inhibitor (Figure 2B).

### Results of Systematic Review

According to the search strategy, A total of 29 patients from 7 families, and 3 sporadic patients harbored different mutations were included (Table 2). Ten *SCNN1G* mutations, including 4 nonsense mutations, 3 missense mutations, and 3 frameshift mutations were found, 3 of them were validated for pathogenicity by patch-clamp monitoring of amiloride-sensitive sodium currents. Except for the patients with missing blood pressure values, all other patients were diagnosed with hypertension. Among included patients, 69% had hypokalemia, 38% had suppressed PAC, and 79% had suppressed PRA. Four patients (14%) had cerebrovascular events.

**TABLE 2:** Results of systematic review of published studies regarding LS patients carrying SCNN1G mutations.

Mutation sites	Pedigree sporadic cases	Subjects	Sex	Onset of age (years)	Stroke	BP (mmHg)	Hypokalemia	Suppression of PAC	Suppression of PRA	Vitro studies	Amiloride-sensitive current
p.Asn530Ser	Family	Case1	M	25	No	180/120	Yes	Yes	Yes	*Xenopus oocytes*	2.0-fold
		Case2	F	40	No	160/105	Yes	NA	Yes		
p.Gln567*	Family	Case1	M	20	No	180/120	Yes	Yes	Yes	NA	NA
		Case2	M	NA	No	NA	NA	NA	NA		
		Case3	M	NA	No	191/118	NA	NA	NA		
p.Glu571*	Family	Case1	F	18	No	216/118	Yes	No	Yes	*CHO* cells	3.7-fold
		Case2	F	31	Yes	176/106	Yes	No	Yes		
		Case3	M	19	Yes	210/120	Yes	No	Yes		
		Case4	F	18	No	140/110	Yes	No	Yes		
		Case5	M	NA	Yes	NA	NA	NA	NA		
		Case6	M	7	No	120/72	Yes	No	Yes		
p.Trp573*	Family	Case1	F	17	No	180/106	Yes	Yes	Yes	*Xenopus oocytes*	7.5-fold
		Case2	F	22	No	150/100	Yes	No	Yes		
		Case3	M	15	No	147/90	No	No	Yes		
		Case4	M	12	No	187/114	Yes	Yes	Yes		
		Case5	F	26	No	160/110	Yes	No	Yes		
		Case6	F	26	No	170/110	Yes	No	Yes		
p.Trp575*	Sporadic	Case1	F	14	No	156/108	Yes	Yes	Yes	NA	NA
p.Glu583Aspfs*585	Sporadic	Case1	M	13	No	NA	Yes	No	Yes	NA	NA
p.Gly590Alafs	Family	Case1	M	12	No	159/109	Yes	No	Yes	NA	NA
		Case2	F	14	No	160/100	No	Yes	Yes		
p.Arg586Valfs*598	Family	Case1	F	NA	No	120/86	No	No	No	NA	NA
		Case2	M	19	No	180/110	No	Yes	Yes		
		Case3	M	14	No	190/120	No	Yes	Yes		
p.Pro625Leu	Family	Case1	M	14	No	160/100	No	No	No	NA	NA
		Case2	F	28	Yes	180/110	Yes	Yes	Yes		
		Case3	M	30	No	180/120	Yes	Yes	Yes		
		Case4	M	3	No	120/80	Yes	Yes	Yes		
p.Pro625Arg	Sporadic	Case1	M	13	No	>140/90	Yes	NA	NA	NA	NA

F, female; M, male; BP, blood pressure; CHO, Chinese Hamster Ovary; PRA, plasma renin activity; PAC, plasma aldosterone concentration. *PRA and *PAC were tested after being kept in a standing position for 2 h; NA, not available.

## Discussion

In this study, the pathogenicity of a nonsense mutation (c.1171 G > T) in *SCNN1G* was confirmed by functional experiments. The blood pressure and electrolyte concentrations of LS patients in this family returned to normal after 1 month of amiloride treatment, and no target organ damage and cardiovascular events occurred over a 7-year follow-up.

LS is a rare autosomal dominant disease, the prevalence reported as 0.91 and 1.52%, respectively (2, 17). LS is caused by mutations in *SCNN1A*, *SCNN1B*, and *SCNN1G*, which encode ENaCs in kidney tubules, affecting sodium reabsorption (18). Proline-rich segments in the C terminals of β and γ subunits, which are critical for function, are known as PY motifs (19). The ubiquitin ligase NEDD4 binds to ENaC PY motifs, leading to the ubiquitination and degradation of ENaC (20). Mutations typically prevent the ubiquitination of subunits, thus inhibiting the rate at which they are internalized from the membrane, resulting in increased ENaC enrichment and elevated channel activity (3). However, a minority of mutations increase ENaC activity by changing the open probability in the membrane (20, 21). In 2017, a *SCNN1A* mutation, a gain-of-function mutation in the extracellular domain of the α subunit, was reported, which primarily increased channel open probability rather than channel surface density (8). By altering ENaC activity, sodium reabsorption increases, which leads to volume expansion and hypertension.

There are 29 mutation sites linked to LS, and nearly all mutations delete or alter PY motifs, and include frameshift, nonsense, or missense mutations (12, 20, 22–24). Among the *SCNN1G* genetic spectrum, 10 sites have been detected (3, 13, 22, 23), including four nonsense mutations, three missense mutations, and three frameshift mutations (Table 2). The first identified *SCNN1G* mutation was a nonsense mutation (p.Trp573*) reported by Hansson et al.; *Xenopus oocytes* expressing mutant p.Trp573* displayed 7.5-fold increase in amiloride-sensitive sodium current than wild-type (25). In the following years, a nonsense mutation site (p.Trp575*) was detected in a sporadic case with hypertension in Japan (26), and Shi et al. and Zhang et al. reported a p.Gln567* mutation in a Chinese family and sporadic case, respectively (27, 28). However, pathogenic functional experiments were not conducted for the p.Gln567* mutation. Three frame mutations and one missense site have been reported in different families, and their clinical features are summarized in Table 2. Most mutation sites impact PY motifs, except for p.Asn530Ser. The p.Asn530Ser mutation is in the extracellular domain and does not influence the PY motif, but it does cause an LS phenotype (21). In patch-clamp experiments, the p.Asn530Ser mutation presented twofold higher amiloride-sensitive currents compared with the wild type, by increasing the open probability of channels (21). The p.Glu571* mutation was first reported by our team in a Chinese family, and this variant has also been identified in another Chinese family (2, 17). In the present functional experiments, cells with the p.Glu571* mutation displayed 3.7-fold amiloride-sensitive currents of wild-type cells.

LS is a kind of channelopathy, and patch-clamp experiments are very important for verifying ion channel diseases. According to previous research, pathogenic mutations in *SCNN1G* mostly affect the PY motif, but pathogenicity has only been functionally verified in three sites. The physiological characteristics of ENaCs expressed in *Xenopus oocytes* are similar to those of ENaCs in human distal renal tubules (29); thus, functional experiments usually use *Xenopus oocytes* for detecting current variations in patch-clamp experiments. In the present study, however, we use *CHO* cells to detect current variations. *CHO* cells are used in patch-clamp electrophysiological experiments because of their low endogenous expression of ion channels (30). Amiloride-sensitive-sodium currents were suppressed after adding amiloride in the present study, indicating drug efficacy from an ion channel aspect. Different variants display different degrees of amiloride-induced sodium current changes, which may be associated with the effects of mutation sites on PY motifs and the cell model. Compared with extracellular domain mutations, PY motif mutations expressed higher amiloride-sensitive currents, indicating that PY motif mutations have a greater impact on ENaC activity.

Phenotypes vary greatly in patients harboring different mutations, as well as for the same mutation in a pedigree (Table 2). A systematic review conducted in 2018 reported that 92.4% of patients with LS presented hypertension (3). In the current pedigree, all members carrying the p.Glu571* variant presented hypertension, and some even had hypertensive stroke. Moreover, one of the typical features of LS is early-onset hypertension; most patients develop hypertension before 30 years. The youngest reported patients are a 10-week-old baby, who was a sporadic case, and a 2-year-old child from an LS family (9, 31). According to the largest retrospective analysis of LS patients, the average onset age of hypertension is 15.5 ± 3.3 years (7). In our research, the index case was diagnosed with hypertension at 18 years old with left ventricular concentric hypertrophy. We speculated that the proband probably had hypertension in child, and she had no obvious symptoms and had not been diagnosed in time. Another 7-year-old pediatric patient had hypertension, hypokalemia, and no target organ damage, as well he had no obvious symptoms and his onset age is unclear. The remaining family members diagnosed with hypertension are almost less than 30 years old.

In this family, there was heterogeneity not only in the occurrence of high blood pressure, but also in the severity of hypertension and complications. Reported complications of persistent hypertension in LS patients include cerebrovascular accidents, early death, renal insufficiency, nose bleeds, and retinal damage (7, 12, 32). Cerebrovascular accidents before the age of 40 were common in this LS family, especially stroke, which causes a heavy burden to patients and their families. The reason for the high incidence of stroke in this family may include poor management of blood pressure, genotype, and environmental factors.

Hypokalemia is present in 71.8% of cases of LS (3). In the current study, all mutation-carrying participants exhibited hypokalemia. Tamura et al. and Fan Peng et al. reached a similar conclusion: that some LS patients have hypertension but exhibit normokalemia (13, 33). The suppression of PRA and PAC is considered a typical presentation, and can be used to differentiate other possible causes of secondary hypertension (34). In our study, all members presented with suppression of PRA, which is in line with the typical characteristics of LS. However, the PAC levels of all members were within the normal range, and members harboring the same variant in another LS pedigree also presented normal PAC (17). The presentation of PAC shows clear heterogeneity compared with other variant-related phenotypes. The reasons for the heterogeneity of LS may be related to aspects including environmental factors and gene polymorphisms. LS patients with *SCNN1A* mutation may presented with mild phenotype than typical LS, which may attribute to different pathogenetic mechanisms (8). Therefore, for patients with suspected LS phenotype but milder symptoms, the possibility of carrying *SCNN1A* mutation should be considered.

Clinical presentations and laboratory findings in LS are heterogeneous, which can hamper its diagnosis. Family history can provide clues, and combined with early-onset hypertension and laboratory results, can be used to identify suspected LS patients. Inadequate symptoms and insufficient awareness of children with monogenic hypertension leading to fail to intervene in time, and most of them have developed complications in youth (12). According to the previous study, 90% pediatric patients had LS family history, so genetic screening can provide definitive confirmation of adult, and pediatric LS (22, 35). Additionally, investigation of the pathogenicity of variants based on amiloride-sensitive currents is needed.

There were several limitations in the present study. This site was not a novel mutation—it has been briefly mentioned in a review by our team. Furthermore, we only compared the amiloride-sensitive sodium currents of γGlu571* mutations with wild-type channels, and not with other truncation mutations of the γ subunit, which may explain the heterogeneous results to some extent. The tailored treatment with amiloride efficacy in blood pressure control and avoiding cardiovascular events has only been validated in the long-term follow-up results of this family, which cannot be representative of all LS patients. A cohort study of LS patients is needed to explore the efficacy of amiloride in improving prognosis and blood pressure control.

## Conclusion

In conclusion, wen identified the pathogenicity of a nonsense mutation by functional analysis. This discovery not only expands the spectrum of *SCNN1G* mutations, but also increases the pathogenicity grades. Tailored treatment with amiloride may be an effective treatment for long-term blood pressure management and preventing cardiovascular events, so timely intervention makes a difference for adult and pediatric patients.

## Data Availability Statement

The original contributions presented in the study are included in the article/supplementary material, further inquiries can be directed to the corresponding author/s.

## Ethics Statement

The studies involving human participants were reviewed and approved by the Fuwai Hospital, National Center for Cardiovascular Diseases. Written informed consent to participate in this study was provided by the participants’ legal guardian/next of kin.

## Author Contributions

DZ, Y-XL, and X-LZ designed the study and modified the manuscript. DZ, YQ, X-QD, C-XY, and X-CL collected clinical information and performed data analysis. DZ, Y-TL, K-QY, PF, YX-H, and L-GG performed experiments. DZ wrote the manuscript. All authors reviewed this work.

## Conflict of Interest

The authors declare that the research was conducted in the absence of any commercial or financial relationships that could be construed as a potential conflict of interest.

## Publisher’s Note

All claims expressed in this article are solely those of the authors and do not necessarily represent those of their affiliated organizations, or those of the publisher, the editors and the reviewers. Any product that may be evaluated in this article, or claim that may be made by its manufacturer, is not guaranteed or endorsed by the publisher.
