# Corrigendum: Pathogenicity and Long-Term Outcomes of Liddle Syndrome Caused by a Nonsense Mutation of SCNN1G in a Chinese Family

**DOI:** 10.3389/fped.2022.961964

**Published:** 2022-06-30

**Authors:** Di Zhang, Yi Qu, Xue-Qi Dong, Yi-Ting Lu, Kun-Qi Yang, Xin-Chang Liu, Peng Fan, Yu-Xiao Hu, Chun-Xue Yang, Ling-Gen Gao, Ya-Xin Liu, Xian-Liang Zhou

**Affiliations:** ^1^Department of Cardiology, National Center for Cardiovascular Diseases, Chinese Academy of Medical Sciences and Peking Union Medical College, Fuwai Hospital, Beijing, China; ^2^Emergency and Critical Care Center, National Center for Cardiovascular Diseases, Chinese Academy of Medical Sciences and Peking Union Medical College, Fuwai Hospital, Beijing, China; ^3^Department of Geriatric Cardiology, Chinese People's Liberation Army (PLA) General Hospital, Beijing, China

**Keywords:** Liddle syndrome, pathogenicity, pediatrics, amiloride-sensitive current, longterm prognosis

In the published article, corresponding author “Xian-Liang Zhou” was assigned the affiliation of “Department of Geriatric Cardiology, Chinese People's Liberation Army (PLA) General Hospital, Beijing, China.” The affiliation of author “Xian-Liang Zhou” should be “Department of Cardiology, National Center for Cardiovascular Diseases, Chinese Academy of Medical Sciences and Peking Union Medical College, Fuwai Hospital, Beijing, China.”

The authors apologize for this error and state that this does not change the scientific conclusions of the article in any way. The original article has been updated.

## Publisher's Note

All claims expressed in this article are solely those of the authors and do not necessarily represent those of their affiliated organizations, or those of the publisher, the editors and the reviewers. Any product that may be evaluated in this article, or claim that may be made by its manufacturer, is not guaranteed or endorsed by the publisher.

